# A dilation-driven vortex flow in sheared granular materials explains a rheometric anomaly

**DOI:** 10.1038/ncomms10630

**Published:** 2016-02-11

**Authors:** K. P. Krishnaraj, Prabhu R. Nott

**Affiliations:** 1 Department of Chemical Engineering, Indian Institute of Science, Bangalore 560012, India

## Abstract

Granular flows occur widely in nature and industry, yet a continuum description that captures their important features is yet not at hand. Recent experiments on granular materials sheared in a cylindrical Couette device revealed a puzzling anomaly, wherein all components of the stress rise nearly exponentially with depth. Here we show, using particle dynamics simulations and imaging experiments, that the stress anomaly arises from a remarkable vortex flow. For the entire range of fill heights explored, we observe a single toroidal vortex that spans the entire Couette cell and whose sense is opposite to the uppermost Taylor vortex in a fluid. We show that the vortex is driven by a combination of shear-induced dilation, a phenomenon that has no analogue in fluids, and gravity flow. Dilatancy is an important feature of granular mechanics, but not adequately incorporated in existing models.

## Abstract (web summary)

Flowing granular materials exhibit fluid-like features, but not all of them can be described by extending fluid mechanics. Here, the authors show vortex flow in a granular layer sheared between coaxial cylinders, and attribute it to the effect that the material moves away from the sheared region.

## Graphical abstract

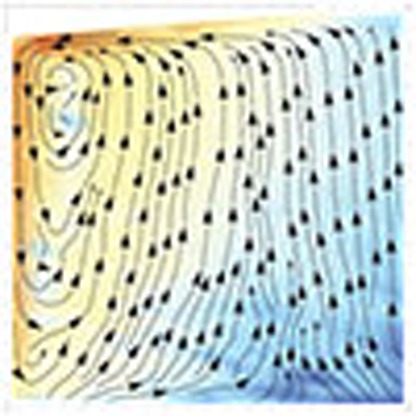

Flowing granular materials exhibit superficial similarities with fluids, but the details of their mechanical response are significantly different, as the micromechanics of grain interactions is quite distinct from that of fluid molecules^1^,^2^. The design of industrial processes and understanding of natural phenomena involving granular flows call for the development of reliable continuum models. Although several advances have been made in this direction^2^,^3^,^4^,^5^,^6^,^7^,^8^,^9^, most models are confined to narrow-flow regimes, or leave out key features. For critical evaluation and refinement of available models, experimental measurement of the rheology is indispensable, for which the protocols of fluid rheometry may be usefully employed.

The rheological properties of fluids are most conveniently measured using devices that generate a class of simple shear flows^10^. The cylindrical Couette cell is such a device, but when it is used for granular materials, unexpected behaviour emerges. Recent studies that used this device for granular rheometry found a striking anomaly in the stress^11^,^12^: the vertical shear stress changes sign on shearing, and the magnitudes of all components of the stress increase roughly exponentially with depth. This behaviour is contrary to previous experiments^13^,^14^ and the predictions of plasticity theories^8^,^13^, which yield a fluid-like stress. After considering several plausible mechanisms, it was speculated^11^,^12^ that an anisotropic microstructure^15^,^16^ is the likely cause of the anomalous stress.

Here we show the cause to be one of the least-expected mechanisms—a single vortex spanning the entire granular column. Vortices arise in fluids too when the rotation rate of the inner cylinder exceeds a critical value, due to the centrifugal Taylor–Couette instability^17^; this instability has also been observed in a fluidized granular bed sheared at high rates^18^. We show that the vortex in a slowly sheared granular material is fundamentally different in its origin and manifestation.

## Results

### DEM simulations and experiments

**Figure 1:**
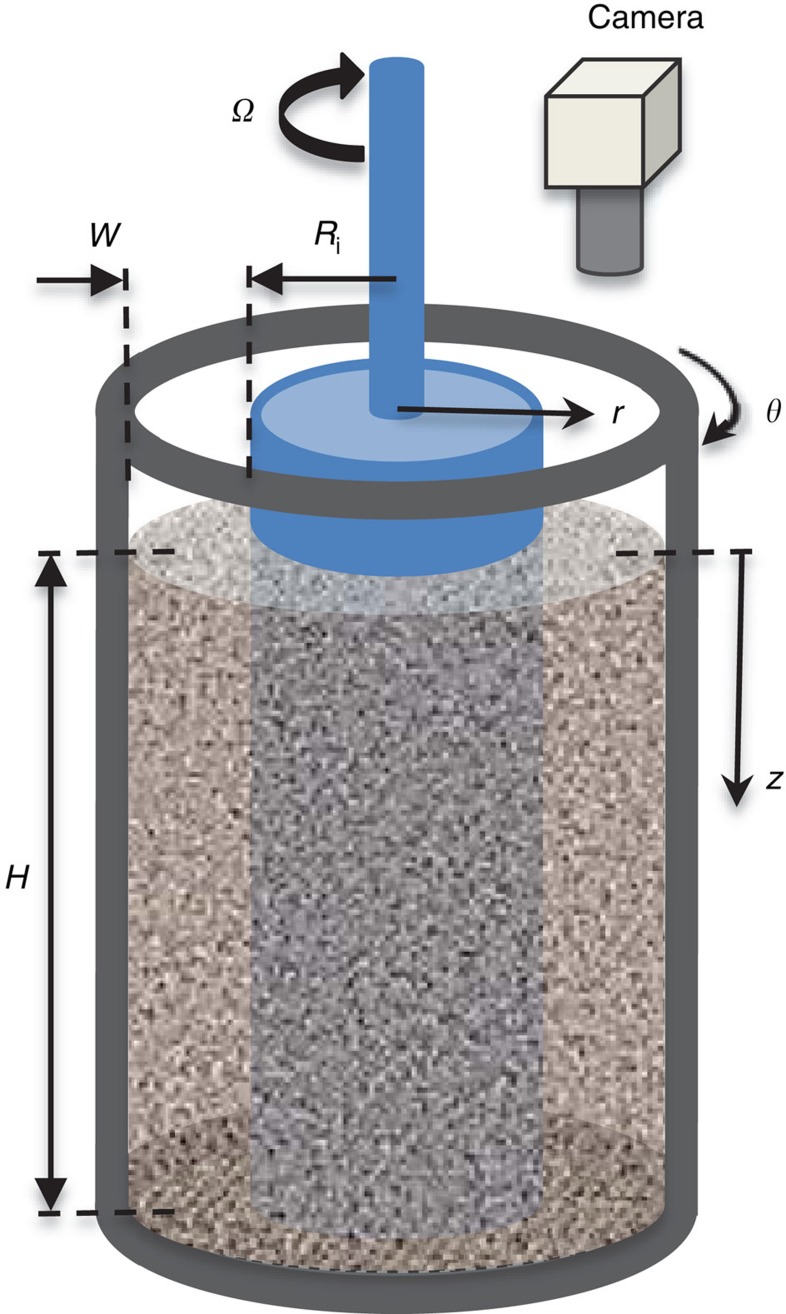
Schematic diagram of the cylindrical Couette cell. The granular material is placed in the annular gap between the two coaxial cylinders of radii *R*_i_ and *R*_i_+*W*, and sheared by rotating the inner cylinder at constant angular speed *Ω*. The DEM simulations are for a Couette cell of dimensions *R*_i_=37 *d*_p_, *W*=16 *d*_p_, and *H*=30, 60 and 90 *d*_p_. The experiments use glass beads of mean diameter *d*_p_=0.83 mm and a Couette cell of dimensions *R*_i_=72 *d*_p_, *W*=18 *d*_p_ and *H*=300 *d*_p_.

To understand the cause of the anomalous stress, we conducted simulations using the discrete element method (DEM)^19^,^20^ (see Methods). The simulations were complemented by video imaging the free surface of an experimental Couette device. In the simulations, the annular gap of a cylindrical Couette cell (Fig. 1) with smooth or rough walls is filled with spherical grains of density *ρ*_p_ and mean diameter *d*_p_ to a fill height *H* by two methods, raining and dense packing. The inner cylinder is then rotated at a constant angular speed *Ω* until a state of steady shear is reached. We varied *Ω* over a range that spans the quasistatic and lower end of the intermediate regimes, which are delineated by the Savage number *Sa*, defined as the ratio of the stress due to impulsive grain collisions to the total stress (Supplementary Note 1). Unless stated otherwise, the DEM results shown in the paper are for smooth walls, filling by raining and *Sa*=2 × 10^−6^; however, the qualitative features of the kinematics and stress are independent of the wall roughness, the Savage number, the parameters in the grain contact model and the method of filling. In the experiments, the steady-state velocity profile at the free surface is determined by video imaging and particle image velocimetry (see Methods).

### Validation of the DEM simulations

**Figure 2:**
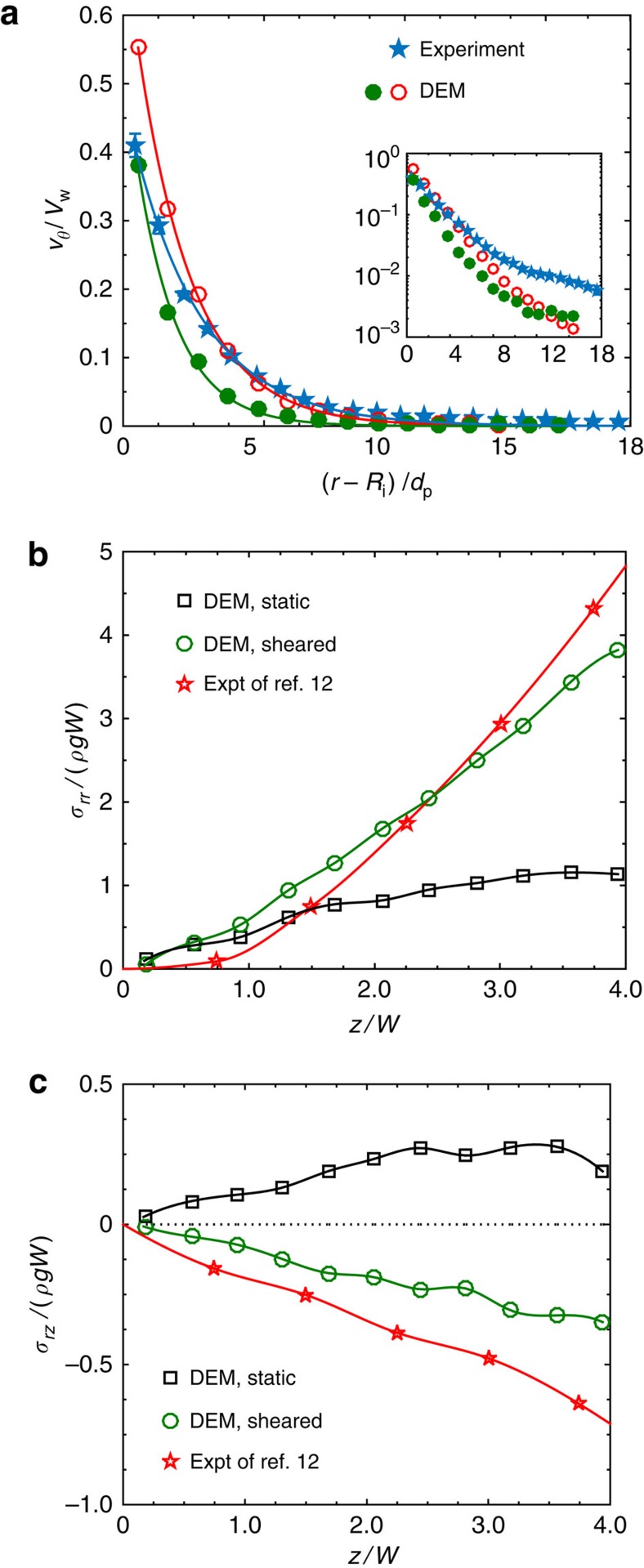
Validation of the DEM simulations. (**a**) The azimuthal velocity *v*_*θ*_ (scaled by *V*_w_ ≡*R*_i_*Ω*) at the free surface as a function of radial distance from the inner cylinder; the inset shows the same plot in linear-log scale. The green filled circles are for rough walls, and the red open circles are for smooth walls. The lines are exponential fits *v*_*θ*_=*v*_0_ exp[−(*r*−*R*_i_)/*β*] of the data, with *β*=2.5 *d*_p_ (experiment), 2 *d*_p_ (DEM, rough walls) and 1.5 *d*_p_ (DEM, smooth walls). The error bars, indicating one standard deviation about the mean, are obtained from eight independent measurements; error bars for the DEM simulations are smaller than the size of the symbols. (**b**,**c**) Variation with depth of the normal stress and the vertical shear stress on the outer cylinder for static and sheared columns with rough walls; here *ρ* is the average bulk density in the Couette cell. The lines are cubic spline fits of the data, and the experimental data are from ref. 12. In all the panels, the DEM results are for *H*=90 *d*_p_.

The results of our DEM simulations are first validated by comparison with experimental data. The kinematics of the azimuthal flow is compared with the data obtained from video imaging (Fig. 2a). The simulations reproduce the exponential decay of *v*_*θ*_ with distance from the inner cylinder, a feature that is characteristic of slow granular flows^21^,^22^,^23^. The mechanics is validated by comparing the stress at the outer cylinder with the data of Gutam *et al*.^12^ (Fig. 2b,c). The simulations reproduce qualitatively all the features of the anomalous stress observed in the experiments: in a static column, the shear stress *σ*_*rz*_ is positive (that is, the vertical component on the traction on the wall is downwards) and all components of the stress saturate asymptotically with the depth *z*, in accord with the classical Janssen solution^24^,^25^ (Supplementary Note 2); on shearing, *σ*_*rz*_ changes sign and the normal stress *σ*_*rr*_ acquires a positive curvature. The quantitative differences between the simulation and experimental data may be partly due to the Couette cell in the simulations being much smaller, for computational tractability; moreover, we do not attempt to tune the parameters of the contact model to achieve agreement, as our primary aim is to gain a qualitative understanding of the cause of the anomalous stress. Nevertheless, our qualitative validation is useful because the features we verify are significant: the exponential decay of *v*_*θ*_(*r*), a characteristic feature of slow granular flows, and the reversal in the sign of *σ*_*rz*_ on shearing, a key finding of our previous experimental studies.

**Figure 3:**
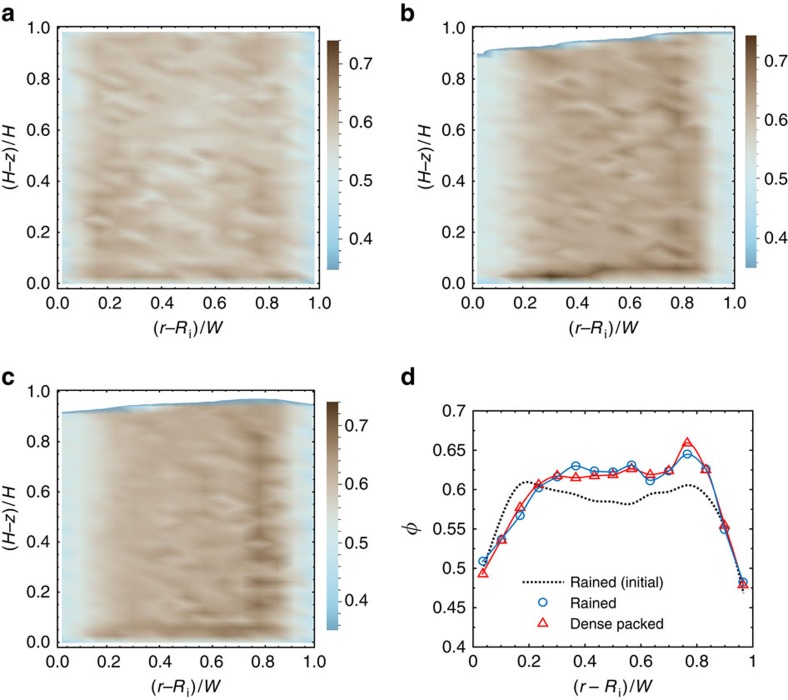
Density distribution and dilation. (**a**–**c**) Distribution of the solids fraction *φ* before commencement of shear after filling by raining (**a**), at the sheared steady state after filling by raining (**b**), and at the sheared steady state after filling by dense packing (**c**). The colours indicate the value of *φ*. (**d**) The radial profile of *φ* at *z*=15 *d*_p_. The results in all the panels are for fill height *H*=30 *d*_p_ and rough walls.

Starting from an initial state generated by raining, shearing results in overall compaction, as seen from the fall of the free surface (Fig. 3a,b) and the profiles of the solids fraction (Fig. 3d), because raining yields a loosely packed initial state. The low-density layers adjacent to the two cylinders are due to the wall-imposed constraints on packing. Nevertheless, significant dilation in the shear layer is evident from the broadening of the low-density layer near the inner cylinder, in accord with numerous experimental observations^2^,^26^. The volume-fraction distributions at steady state for the two filling methods are almost identical (Fig. 3b–d), leading us to the conclusion that the initial preparation of the granular bed has little bearing on the properties at steady state.

### The secondary vortex

**Figure 4:**
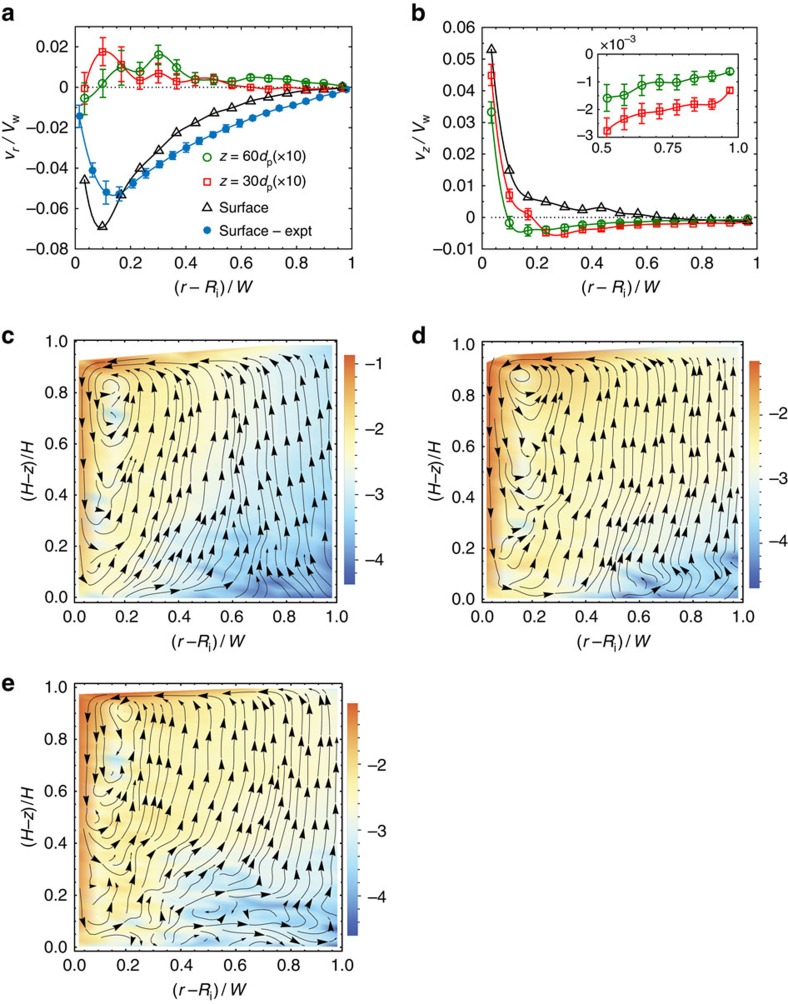
The form of the secondary flow. (**a**,**b**) The velocities in the radial and vertical directions as a function of *r* at different *z*. The open symbols are DEM results for *H*=90 *d*_p_ and the filled circles are experimental data. The error bars are obtained in the manner described in the caption of Fig. 2. (**c**–**e**) Streamlines of the secondary flow at steady state for fill heights *H*=30 *d*_p_ (**c**), 60 *d*_p_ (**d**) and 90 *d*_p_ (**e**). The background colour indicates the value of log_10_ *s*, where 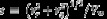 is the magnitude of the secondary flow.

Further examination of the kinematics reveals a secondary flow in the *r*-*z* plane, superimposed over the azimuthal flow. Although its velocity scale is small compared to that of the azimuthal flow, the radial and vertical velocities *v*_*r*_ and *v*_*z*_ are easily measurable, and show a systematic trend (Fig. 4a,b). The shapes of the *v*_*r*_ profiles at the free surface determined from experiment and DEM simulations are in good agreement. When the velocities at all the locations are combined to construct the streamlines, a single vortex that extends over the entire width and height of the Couette gap emerges (Fig. 4c; Supplementary Movie 1). Two aspects of the secondary flow are notable: The first is that a thin layer adjacent to the free surface flows radially inwards (Supplementary Movie 2). In contrast, the Taylor vortex in a fluid causes an outward radial flow near the free surface, if the fluid column is shallow enough to have only one vortex^27^. Second, there is always a single vortex for the range of fill heights *H* we have studied (Fig. 4c–e), whereas the size of Taylor vortices scales with *W*, resulting in a vertical train of counter-rotating vortices. The qualitative features of the vortex are independent of the Savage number and the wall roughness (Fig. 4e; Supplementary Fig. 1a–c).

We note that the free surface slopes downward from the outer to inner cylinders (Fig. 3b,c), a feature that is also observed in the experiments; while the time taken for the free surface slope and the secondary flow to reach steady state roughly coincide, our evidence is insufficient to infer whether the slope is caused by the secondary flow, or another aspect of the mechanics (such as normal stress differences^10^).

### The vortex explains the anomalous stress

To show that the vortex flow explains the anomalous stress, we consider the simplest plasticity model for the stress tensor ***σ***^28^,^29^,


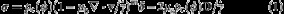


where **v** is the velocity vector, **D** the deviatoric part of the deformation rate tensor (with scalar norm 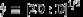), ***δ*** the identity tensor, and *p*_c_(*φ*) is the pressure at the critical state^2^,^5^. The parameters in the model are the bulk and shear plastic moduli, *μ*_b_ and *μ*_s_, and the exponent m. The above is a model for rate-independent plasticity if *μ*_b_ and *μ*_s_ are independent of 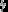, and rate-dependent plasticity otherwise^9^,^30^. Applying the model to the problem at hand, at the two cylinders impenetrability requires *v*_*r*_=0, whence *D*_*rz*_=∂*v*_*z*_/∂*r*. From the radial variation of *v*_*z*_, we see that ∂*v*_*z*_/∂*r*>0 at the outer cylinder (inset of Fig. 4b). Equation (1) then implies that *σ*_*rz*_<0, which is in accord with experimental observations^11^,^12^ (Fig. 2c). Thus, the vortex explains the reversal in the sign of *σ*_*rz*_ when the granular column is sheared. This result, in conjunction with Coulomb friction at the boundaries, explains the sharp rise of all components of the stress with *z*^11^,^12^ (Supplementary Note 2; Supplementary Fig. 2), and thereby all aspects of the anomalous stress. Although we have used a simple plasticity model to illustrate the link between the vortex and the anomalous stress, more elaborate models^2^,^29^ lead to the same conclusion.

### Mechanism for the secondary vortex

**Figure 5:**
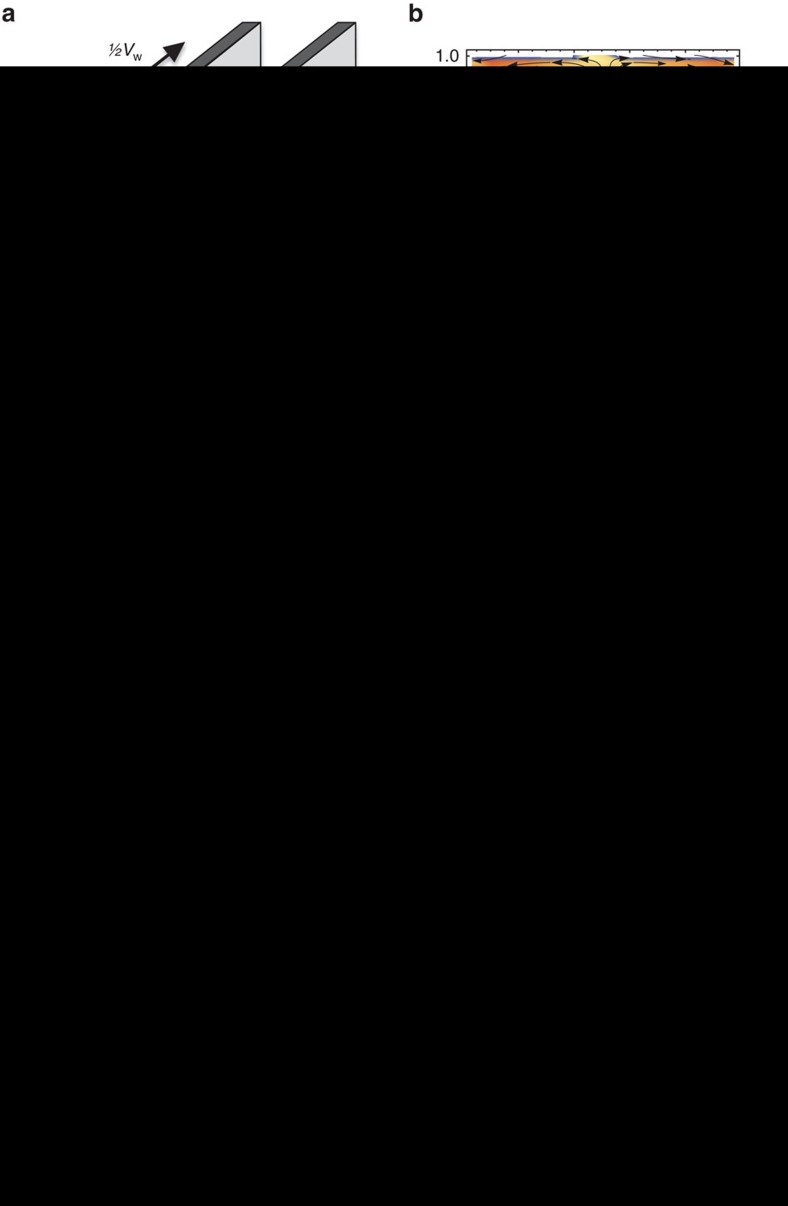
The cause of the secondary vortex. (**a**) Schematic diagram of plane shear between vertical walls. (**b**) Streamlines of the secondary flow in plane shear. The unit cell of dimensions *W*=16 *d*_p_, *H*=30 *d*_p_ and *L*=50 *d*_p_ is replicated periodically in the *x* direction. (**c**–**e**) Transient evolution of the secondary flow in a cylindrical Couette cell after initiation of shear: the streamlines after an inner cylinder rotation of 3.4° (**c**), 22° (**d**) and 45° (**e**). The background colour indicates the value of log_10_ *s*, where 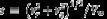 is the magnitude of the secondary flow. In **c**–**e** the Couette cell is filled by the dense packing method to a fill height *H*=30 *d*_p_ and the walls are rough; the velocity fields are averaged over the preceding 3.4° in (**c**) and 8° in (**d**,**e**).

We now address the cause of the secondary flow. The sense of the vortex indicates that it is not centrifugal in origin, but for a definitive confirmation, we conduct a simulation of shear between two plane vertical walls (Fig. 5a). Figure 5b shows the secondary flow that results—two counter-rotating vortices placed symmetrically about the mid-plane of the Couette gap (see also Supplementary Movie 3). Symmetry arises here because the two walls are indistinguishable, unlike in cylindrical Couette flow. Thus, the vortices closely resembling those in Fig. 4c–e, arise in the complete absence of the centrifugal force.

What then drives the vortex? This question is answered by considering the transient evolution of the secondary flow soon after initiation of shear. The streamlines in Fig. 5c–e show that the flow is initially radially outwards and upwards, and concurrently, there is downward flow close to the inner cylinder. These two flows combine to curl the streamlines towards the inner cylinder at later time, eventually establishing a steady vortex (Fig. 4c) within a 90° rotation of the inner cylinder. When gravity is turned off and a frictionless wall used to confine the material from the top, we find no secondary vortex. It is now clear that the vortex is driven by the combination of shear-induced dilation and gravity flow—dilation causes the material to flow away from the shear layer and ultimately to the free surface, and the downward flow of grains near the inner cylinder sustains the vortex. The sense of the vortex is determined by the asymmetry in dilation between the inner and outer cylinders (Fig. 3), as a result of the shear rate of the primary flow decaying exponentially with radial distance from the inner cylinder (Fig. 2a).

We have used the transient evolution of the vortex to elucidate the mechanism, but it applies equally well at steady state. In the absence of gravity flow, dilation would cease after the initial transient, and a steady state would be reached wherein the flow is purely azimuthal and there is a radial density gradient. While gravity flow is essential to sustain the vortex, we surmise that it is initiated by dilation, as we cannot think of another physical mechanism that would cause a radial flow.

Importantly, we find the secondary vortex to be present even when the upper surface is not traction-free. Supplementary Figure 3 shows the streamlines at steady state for plane Couette flow when the material is confined at the top by a horizontal rigid plate of fixed weight, which is allowed to move vertically. The presence of two counter-rotating vortices is evident, as in the case of a traction-free surface (Fig. 5b), though their strength and symmetry decrease as the weight of the confining plate increases. We find the vortex to persist in cylindrical Couette flow too when confined at the top, and its basic structure is largely independent of the confinement condition and plate roughness (K.P.K., P. V. Dsouza, T. Murthy and P.R.N., manuscript in preparation). Thus, a traction-free surface is not essential for the formation of the vortex—the combined effects of dilation and gravity break the up–down symmetry, and determine the sense of the vortex.

## Discussion

The dilation-driven secondary flow that we report is a novel phenomenon that has no analogue in fluids. Although the scale of the secondary flow is smaller than the primary azimuthal flow, its rheological signature is large, owing to the dependence of the shear stresses on the pressure (equation (1)). Evidence of a secondary flow was provided by two previous studies^31^,^32^, the former hypothesizing dilatancy as a possible cause and the latter finding convection to vanish under microgravity conditions; however, they could not discern the detailed form of the secondary flow and its origin. Our results establish that there is a single vortex (for the range of fill heights we have explored), resulting from the combined effects of dilatancy and gravity flow.

It has been known for long that dilation accompanies deformation in dense granular materials, and, conversely, compaction when the material is loose^2^,^33^. This key feature distinguishes plastic deformation of granular materials from that of metals, and of course fluids. Existing rheological models for granular flows are inadequate for capturing compressibility; while density change along a streamline is predicted (such as in flow through a hopper), to our knowledge no model captures the shear-induced density gradient across streamlines^8^. It is usually assumed that the effect of dilatancy is confined to the (typically) narrow shear layer, but our study establishes that it can act as a driving force for a large-scale flow. Thus, despite some advances, much remains to be done towards formulating a robust and effective rheological model for granular materials—we expect that our study will provide a useful impetus in this direction.

The Taylor vortex in fluids arises from an instability of the base state of azimuthal flow, due to an imbalance between the centrifugal force and the radial pressure gradient^17^. It is quite possible that the granular vortex arises from a similar hydrodynamic instability, with dilation as the driving force. However, a stability analysis must await the formulation of a rheological model that captures cross-streamline dilation. We finally note that the anomalous stress reported by our group^11^,^12^ was then ascribed to microstructural anisotropy; while we now offer a more compelling explanation, there is sufficient evidence in the literature to motivate the incorporation of an anisotropic fabric in a comprehensive rheological model.

## Methods

### DEM simulations

DEM is a widely used computational tool for granular mechanics, where the positions and interactions of all the particles are tracked in time using simple models for grain interactions. We used the soft-particle contact model^19^,^20^, wherein the grains are treated as deformable spheres, but rather than calculate their deformation in detail, they are allowed to overlap. The interaction forces are written in terms of the overlap and its time rate of change. The normal and tangential forces have elastic and viscous components, and the tangential force incorporates an additional Coulomb slider to capture rate-independent friction (Supplementary Fig. 4a). Considering the contact of grains *i* and *j* centred at position vectors **r**_*i*_ and **r**_*j*_, the normal and tangential forces on particle *i* are^34^


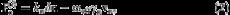



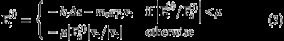


respectively. Here *δ*≡*R*_*i*_+*R*_*j*_−|**r**_*i*_−**r**_*j*_| is the overlap, **n** is the unit normal at the point of contact pointing towards the centre of *i*, **v**_n_ and **v**_t_ are the relative velocities at the point of contact in the normal and tangential directions, respectively, Δ**s** is the tangential displacement and *m*_eff_ ≡(1/*m*_*i*_+1/*m*_*j*_)^−1^ is the effective mass that determines the damping force. The spring stiffness constants *k*_n_, *k*_t_, the damping constants *γ*_n_, *γ*_t_ and the Coulomb friction coefficient *μ* are the parameters that define the interaction. The motion of each particle is determined by integrating Newton's second law, assuming pairwise additivity of the interaction forces. The computationally intensive simulations, involving the tracking of ∼5 × 10^5^ particles for durations corresponding to several rotations of the inner cylinder, were conducted using the LAMMPS package^35^.

To avoid crystalline order, a mixture of grains of diameter 0.9 *d*_p_, *d*_p_ and 1.1 *d*_p_ (of mass fractions 0.3, 0.4 and 0.3, respectively) was used. Simulations were conducted for two types of boundaries (cylinders and bottom wall): the surfaces were either smooth, or coated with a rigid, close-packed triangular lattice of grains of diameter 0.9 *d*_p_; the two cases are referred to as smooth and rough walls, respectively. For both types of walls, the grain–wall interactions were computed by treating the walls as bodies of infinite mass, but with the same stiffness, damping and friction constants as for grain–grain interactions.

For values of the spring constants *k*_n_ and *k*_t_ that are appropriate for hard particles like sand and glass beads, accurately resolving each contact requires so small a time step of integration that the computation time becomes prohibitively high. It is therefore standard practice to optimize their values such that they are low enough for the computations to be tractable, but high enough that the macroscopic behaviour mimics that of hard particles. The values chosen for the simulations are *k*_n_=10^6^*m*_p_*g*/*d*_p_, *k*_t_=2/7*k*_n_, as per previous studies that have modelled hard grains^16^,^34^. Here *m*_p_ is the mass of the particle of diameter *d*_p_ and *g* is the gravitational acceleration. The damping coefficients were set to 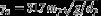, *γ*_t_=1/2*γ*_n_ and the friction coefficient to *μ*=0.5. The chosen value of *γ*_n_ yields a normal coefficient of restitution for a collision of 0.7 (ref. 16). To verify that the results do not depend qualitatively on the parameter values^36^, simulations were conducted for three values of *k*_n_ varying over two decades. The profiles of the normal and vertical shear stress at the outer cylinder are shown in Supplementary Fig. 4b,c. Increasing *k*_n_ results in larger magnitudes of the stress components, but the qualitative features that characterize the stress anomaly remain independent of *k*_n_.

The Couette cell was filled by two methods, raining and dense packing. In the former, the mixture of grains (of sizes mentioned above) was poured uniformly over the annular gap from a reservoir at the top of the Couette cell, until the fill height *H* was reached. In the latter, grains of uniform size 1.1 *d*_p_ were placed in a body-centred cubic lattice within the Couette cell up to a fill height *H*, in the absence of gravity; the grains were then randomly shrunk to achieve the required size distribution, after which gravity was turned on. The two methods gave an initial average volume fraction of 〈*φ*〉=0.576 and 0.602, respectively.

The continuum variables were obtained by averaging over space and time. The velocity and volume fraction at coordinates (*r*, *z*) were obtained by averaging over all the particles in an annular cylinder of thickness Δ*r*=*d*_p_ (centred at *r*) and depth Δ*z*=3 *d*_p_ (centred at *z*); the wall stresses were obtained by averaging over all particle–wall contacts in such a cylinder adjacent to the outer cylinder. The steady-state variables (Figs 2, 3, 4 and 5b) were time-averaged for half a period of rotation of the inner cylinder, but the transient velocity fields were averaged over much shorter durations (see caption of Fig. 5).

### Experiments

The experiments used spherical beads of soda-lime glass of density *ρ*_p_=2,500 kg m^−3^ and a narrow size distribution with mean diameter *d*_p_=0.83 mm. The Couette apparatus (dimensions in caption of Fig. 1) was filled with the glass beads, and the inner cylinder rotated at 2 revolutions per minute (corresponding to a Savage number *Sa*=3.3 × 10^−6^) for a period of about 30 min to reach steady state. Video images of the free surface were then acquired using a digital video camera (Prosilica GE680) at a rate of 200 frames per second and resolution 640 × 480 pixels (scale factor 0.112 mm per pixel). The instantaneous velocity field was determined by cross-correlation of the intensity maps (averaged over bins of 12 × 12 pixels) in successive frames using the PIVlab^37^ toolbox in MATLAB. The steady-state velocity profile was determined by time-averaging the instantaneous velocity field for 30 s.

## Additional information

**How to cite this article:** Krishnaraj, K. P. & Nott, P. R. A dilation-driven vortex flow in sheared granular materials explains a rheometric anomaly. *Nat. Commun.* 7:10630 doi: 10.1038/ncomms10630 (2016).

## Supplementary Material

Supplementary Information Supplementary Figures 1-4, Supplementary Notes 1-2 and Supplementary References

Supplementary Movie 1 Animation of particle motion in the r-z plane in a DEM simulation of cylindrical Couette flow. The thick black boundaries on the left, right and bottom are the inner cylinder, outer cylinder, and the stationary base, respectively; the sloping boundary at the top is the free surface. The particles in the entire Couette cell are projected onto the plane θ=0, with their r and z coordinates preserved. For clarity of viewing, the particles are rendered at 1/5 of their actual size, and only a small fraction of particles are made visible, which are initially in two layers: one of thickness 3*d*_*p*_ at the free surface, and the other of thickness 2*d*_*p*_ mid-way between the free surface and the base. The particles are colored according to their initial positions, to highlight visualization of the secondary flow—the coloring has no significance otherwise. The time span of the movie corresponds to 3 revolutions of the inner cylinder. The simulation is for fill height H =30*d*_*p*_, Savage number Sa =2×10^-6^, and rough walls.

Supplementary Movie 2 Video image of the free surface of the experimental Couette device. Only a small section of the free surface (of width≈24*d*_*p*_) is imaged. The rotating inner cylinder is at the top of the frame, and the stationary outer cylinder at the bottom. The 15s long movie is in real time. The radial inward flow of the particles is clearly seen.

Supplementary Movie 3 Animation of particle motion in the y-z plane in a DEM simulation of plane Couette flow. The thick black boundaries on the left and right are the two moving walls (see Fig. 5a), and the boundary at the bottom is the stationary base. All the particles in the unit cell are projected onto the plane x=0, with their y and z coordinates preserved. The left and right walls move into and out of the plane, respectively (Fig. 5a). For clarity of viewing, the particles are rendered at 1/5 of their actual size, and only a small fraction of particles are made visible, which are initially in two layers: one of thickness 3*d*_*p*_ at the free surface, and the other of thickness 2*d*_*p*_ mid-way between the free surface and the base. The particles are colored according to their initial positions, to highlight visualization of the secondary flow—the coloring has no significance otherwise. The time span of the movie corresponds to each wall translating a distance of 150*d*_*p*_. The simulation is for fill height *H* =30*d*_*p*_, Savage number Sa =2×10^-6^, and rough walls.
