# Supplementary material for: A dilation-driven vortex flow in sheared granular materials explains a rheometric anomaly
Source: Nat Commun. 2016 Feb 11;7:10630. doi: 10.1038/ncomms10630 (PMC4755268; doi:10.1038/ncomms10630)
Supplement: Supplementary Information — Supplementary Figures 1-4, Supplementary Notes 1-2 and Supplementary References [file ncomms10630-s1.pdf]

Supplementary Information

Supplementary Figure 1

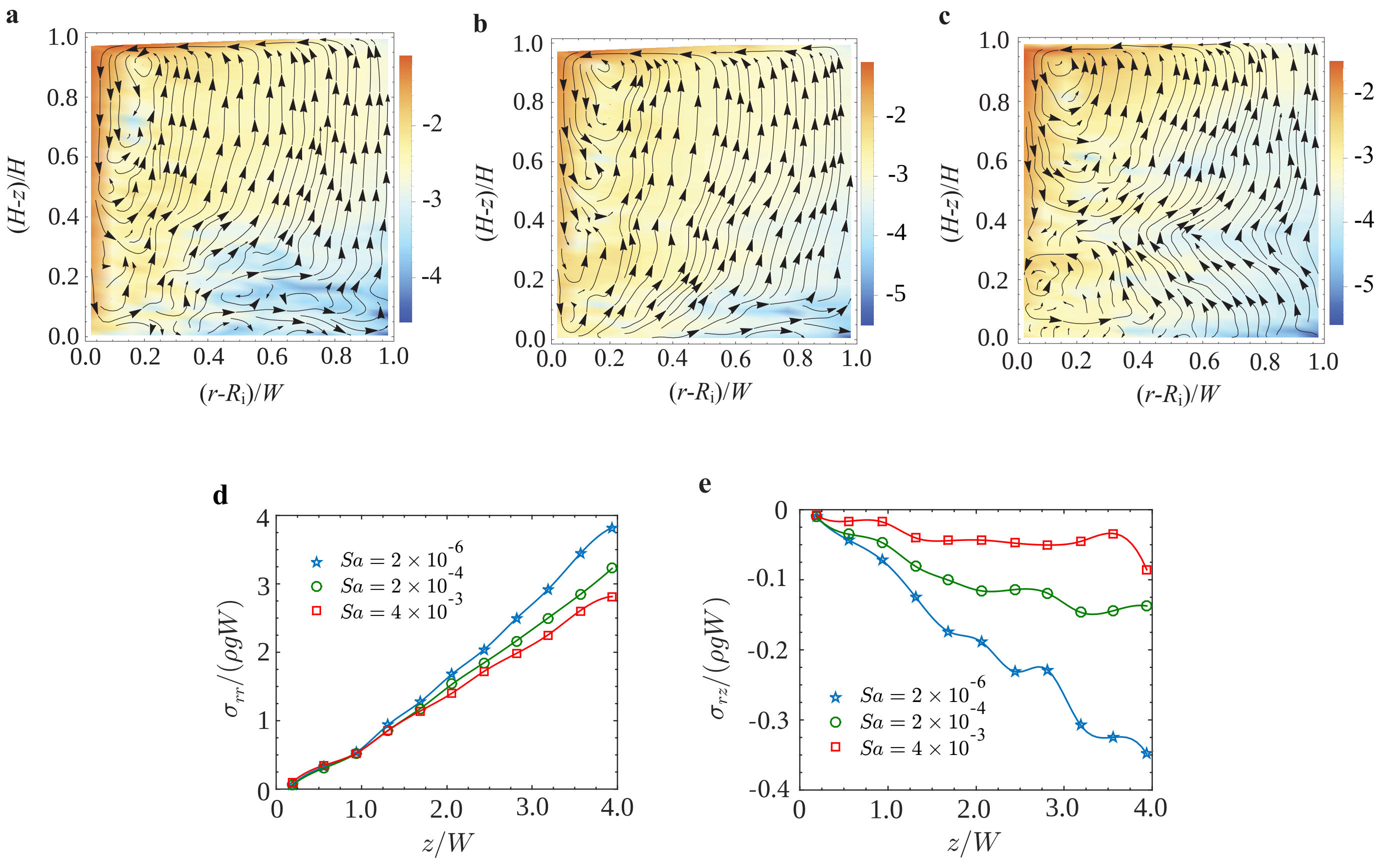

Supplementary Figure 1: Effect of the Savage number on the secondary flow. (**a-c**) The streamlines of the secondary flow for three values of the Savage number, $Sa = 2{\times}10^{-6}$ (panel **a**), $2{\times}10^{-4}$ (panel **b**), and $4{\times}10^{-3}$ (panel **c**). (**d**, **e**) Profiles of the normal stress and vertical shear stress at the outer cylinder for the three values of Sa. The results are for fill height $H = 90\, d_{\mathrm{p}}$ and rough walls, and are discussed in Supplementary Note 1.

Supplementary Figure 2

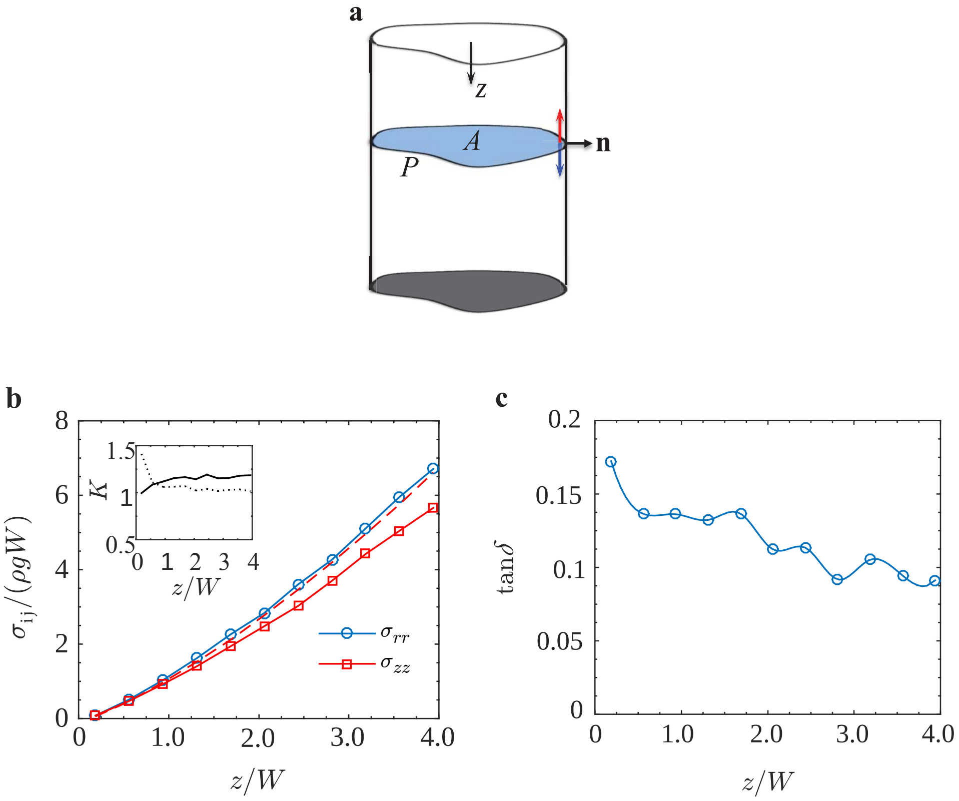

Supplementary Figure 2: Test of the modified Janssen solution. (**a**) Schematic of a vertical container of arbitrary cross section; the cross section area is A, perimeter is P, and $\mathbf{n}$ is the unit normal to the wall. The blue arrow indicates the direction of the vertical traction on the wall when the granular material is static or flowing downwards, and the red arrow indicates the direction of the vertical traction when the flow is upwards. (**b**) The variation of the lateral and vertical normal stresses at the wall with z in the cylindrical Couette device during shear. The solid lines are the perimeter-averaged stresses, and the red dashed line is the cross-section averaged stress $\langle\sigma_{zz}\rangle$. The inset gives the ratios σ_{rr}/σ_{zz} (solid line) and $K = \sigma_{rr}/\langle\sigma_{zz}\rangle$ (dashed line) — note that both are nearly independent of z. (**c**) The variation of the wall angle of friction δ with z. The results in panels (**b**) and (**c**) are for fill height $H = 90\, d_{\mathrm{p}}$, Savage number $Sa = 2{\times}10^{-6}$, and rough walls, and are discussed in Supplementary Note 2.

Supplementary Figure 3

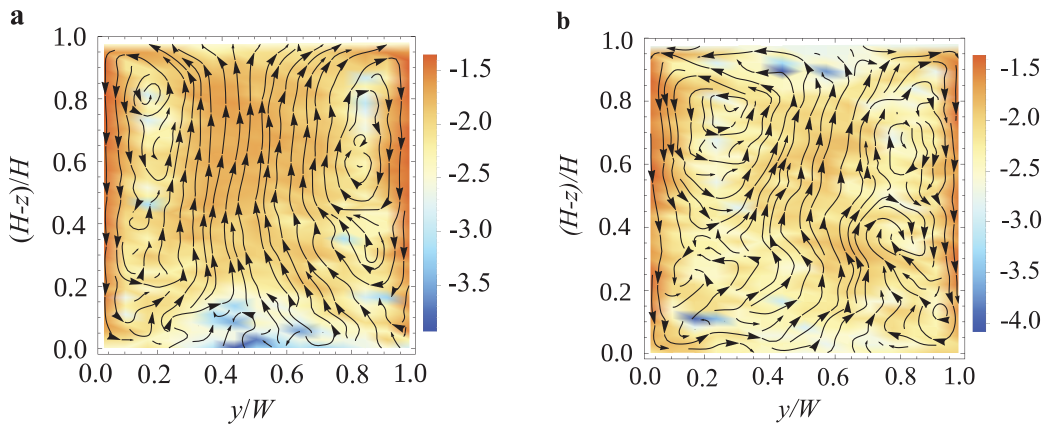

Supplementary Figure 3: Secondary flow with confinement at the top. (**a,b**) Streamlines of the secondary flow for plane Couette flow (Fig. 5a) when the granular material is confined at the top by a rigid plate of weight $0.3\rho g W^2$ (panel **a**) and $\rho g W^2$ (panel **b**) per unit length in the x direction. The plate spans the gap between the two vertical walls, but is allowed to move in the vertical direction without resistance from the walls. The results are for fill height $H = 30\, d_{\mathrm{p}}$, Savage number $Sa = 2{\times}10^{-6}$, and rough walls. The interactions parameters for contact between the grains and the top plate are assumed to be equal to that for grain-grain contact.

Supplementary Figure 4

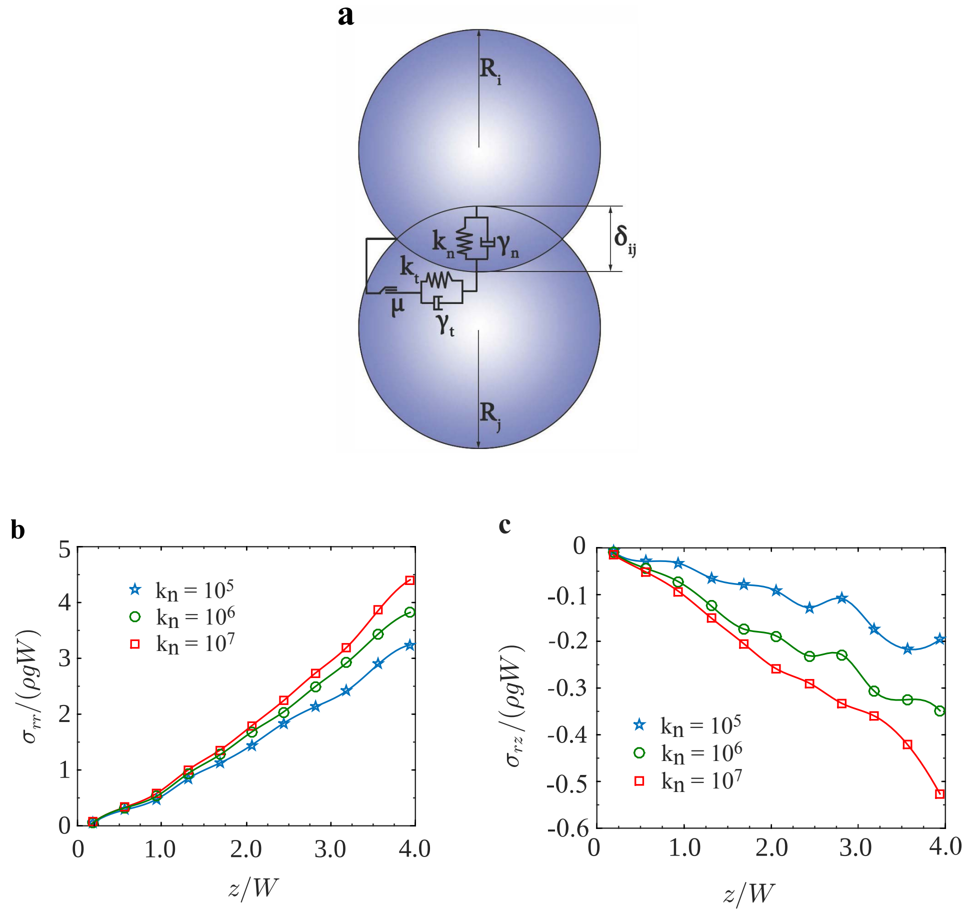

Supplementary Figure 4: Sensitivity of the stress to the spring stiffness. (**a**) Schematic diagram of the soft particle interaction model between spheres of radii R_i and R_j. The stiffness of the normal and tangential springs are k_n and k_t, respectively (see Methods). (**b**, **c**) The normal stress σ_{rr} and the vertical shear stress σ_{rz} at the outer cylinder as a function of depth for three values of the spring stiffness k_n. The values of k_n in the legend are in units of $m_\mathrm{p}\, g/d_\mathrm{p}$. The results are for fill height $H = 90\, d_\mathrm{p}$, Savage number $Sa = 2{\times}10^{-6}$, and rough walls.

Supplementary Note 1: Flow regimes

Granular flows have been mostly studied in the limiting regimes of slow flow (quasistatic) and rapid flow. In the former, grain contacts are sustained, Coulomb friction plays a dominant role, and the stress is roughly independent of the magnitude of the shear rate $\dot{\gamma}$. In the rapid flow regime, grain contacts are impulsive and inelastic, and the stress varies as $\dot{\gamma}^2$. The vast regime lying between these two extremes is loosely termed the intermediate regime, for which the micromechanics of grain interaction is not clearly understood. The flow regimes are characterized by the Savage number (Supplementary Ref.[1]), defined as the ratio of the stress due to grain inertia and the total stress,

$$Sa \equiv \frac{\rho\, d_{\rm p}^2 \dot{\gamma}^2}{N} \tag{1}$$

where ρ is the bulk density, and N is the stress scale. More recent studies[9] (Supplementary Ref.[2]) have introduced the inertia number, which is simply $Sa^{1/2}$. In the problem we consider, the nominal shear rate is $\dot{\gamma} = \Omega R_{\rm i}/W$ (Fig. 1), and N is the Janssen stress scale[2,11] $\rho g W$ (see Supplementary Note 2). The flow is in the quasistatic regime when $Sa <\sim 10^{-6}$, and in the inertial regime when $Sa \sim 1$[9] (Supplementary Ref.[3]). Figures 2–5 are for $Sa = 2\times10^{-6}$; the effect of varying Sa between the quasistatic and lower end of the intermediate regimes is shown in Supplementary Fig. 1. It is clear that the qualitative features of the secondary flow and the anomalous stress remain intact in this range of Sa, but the strengths of the vortex flow and the anomalous stress are considerably reduced at the largest Sa.

Supplementary Note 2: The Janssen and modified Janssen solutions

Static column and downward flow

An analysis of the stress profile for a granular column that is either static or flowing downwards in a fully developed state (i.e., without variation of any quantity in the flow direction) was first presented by Janssen[26,27], and refined later by others (Supplementary Refs[4,5]). For the convenience of the reader, we present below a brief account of the analysis of ref. 2 for a column of arbitrary cross section.

Consider a vertical column of granular material in a container of cross section area A and perimeter P (Supplementary Fig. 2a). The condition of mechanical equilibrium is $\nabla\cdot\boldsymbol{\sigma} = \rho\, g\, \mathbf{e}_z$, where $\mathbf{e}_z$ is the unit normal in the z direction. Integrating its z component over the cross section, and using the

divergence theorem, we get

$$\frac{\partial}{\partial z}\int_A \sigma_{zz}\,\mathrm{d}S + \oint_\ell \sigma_{nz}\,d\ell = \rho\, g A \tag{2}$$

where dS and dℓ are differential elements of the area and perimeter of the cross section, $\mathbf{n}$ is the unit outward normal to the boundary in the plane of the cross section, and $\sigma_{nz} \equiv \mathbf{n}\boldsymbol{\cdot}\boldsymbol{\sigma}\boldsymbol{\cdot}\mathbf{e}_z$ is the vertical shear stress on the container. We now employ the wall friction boundary condition

$$\sigma_{nz} = \sigma_{nn}\tan\delta, \tag{3}$$

where $\sigma_{nn} \equiv \mathbf{n}\boldsymbol{\cdot}\boldsymbol{\sigma}\boldsymbol{\cdot}\mathbf{n}$ is the normal stress on the boundary and δ is the wall angle of friction, usually assumed to be an intrinsic property of the wall and the granular material. Defining $\hat{\sigma}_{nn} \equiv \frac{1}{P}\oint_\ell \mathbf{n}\boldsymbol{\cdot}\boldsymbol{\sigma}\boldsymbol{\cdot}\mathbf{n}\,\mathrm{d}\ell$ as the perimeter-averaged normal stress (and similarly $\hat{\sigma}_{nz}$), and $\langle\sigma_{zz}\rangle \equiv \frac{1}{A}\int_S \sigma_{zz}\,\mathrm{d}S$ as the cross section-averaged vertical normal stress, Supplementary equation (2) takes the form

$$\frac{\mathrm{d}\langle\sigma_{zz}\rangle}{\mathrm{d}z} + \frac{P}{A}\tan\delta\,\hat{\sigma}_{nn} = \rho\, g. \tag{4}$$

Hereafter, we drop the caret (ˆ) over σ_{nn} and σ_{nz} for the sake of convenience, and for conformity with the notation in the paper. For closure, the assumption

$$\sigma_{nn} = K\langle\sigma_{zz}\rangle \tag{5}$$

is made (Supplementary Ref.[5]), where K is the so-called Janssen constant[2]. Substituting the above in Supplementary equation (4) and solving for $\langle\sigma_{zz}\rangle$ using the boundary condition of vanishing stress at the free surface ($z\!=\!0$), we obtain the Janssen solution

$$\langle\sigma_{zz}\rangle = \rho g L\left(1 - e^{-z/L}\right), \quad \sigma_{nn} = K\langle\sigma_{zz}\rangle, \quad \sigma_{nz} = \sigma_{nn}\tan\delta, \tag{6}$$

where $L \equiv A/(PK\tan\delta)$ is the length scale over which the stress saturates; for the cylindrical Couette geometry, $L = (R_\mathrm{i} + R_\mathrm{o})/(2K\tan\delta)$. The physical origin of the saturation of the stress comes from the ability of granular materials to impart a downward vertical traction to the wall by Coulomb friction, even in the absence of flow. Beyond a depth of a few multiples of L, the entire incremental weight of the column is transmitted to the walls through the shear stress σ_{rz}, thus leading to saturation of the stress.

Upward flow

When there is upward flow of the material near a wall, as in the secondary vortex in the present study or when the material is pushed upwards by a piston (Supplementary Ref.[6]), the gradient of the

vertical velocity normal to the wall determines the shear stress σ_{nz}: the simple plasticity model of equation (1) gives

$$\sigma_{nz} = -\frac{\mu_s p_c(\phi)}{\dot{\gamma}} \frac{\partial v_z}{\partial x_n},$$

where x_n is the coordinate perpendicular to the wall, which in the cylindrical Couette cell is the radial coordinate r. Since $\partial v_z/\partial x_n > 0$, we see immediately that $\sigma_{nz} < 0$, i.e., there is an upward traction imposed by the granular material on the wall. Following the spirit of the Janssen solution, Supplementary equation (3) must be modified as

$$\sigma_{nz} = -\sigma_{nn} \tan\delta, \tag{7}$$

where δ is assumed to be constant. Proceeding in exactly the same way, we arrive at the modified Janssen solution[12],

$$\langle\sigma_{zz}\rangle = \rho\, gL \left(e^{z/L} - 1\right), \quad \sigma_{nn} = K\langle\sigma_{zz}\rangle, \quad \sigma_{nz} = -\sigma_{nn}\tan\delta, \tag{8}$$

Thus, the reversal in the sign of the vertical shear stress causes the magnitudes of the normal and shear stresses to rise exponentially with depth, which was the main finding of refs 11,12.

We now evaluate the assumptions in the above analysis that ρ, δ and K are constants. The assumption of constant ρ is a reasonable one, as gravity-consolidation brings the density to close to a constant. The assumption of constant δ is widely made when there is plastic yield (flow) at the wall, or if there is incipient plastic yield (static) — the former is generally valid when the flow is unidirectional (upwards or downwards). The assumption of constant K is formally valid only when the column is static[2] (Supplementary Ref.[7]), but studies have shown that the Janssen solution gives a reasonable estimate of the stress for a static or downward flowing column (Supplementary Refs[7,8]), and similarly the modified Janssen solution for upward flow (Supplementary Ref.[6]). For the problem of cylindrical Couette flow that this paper addresses, the secondary vortex creates a complex flow that is not unidirectional; hence the values of K and δ depend on the components of velocity gradient (see equation 1). Interestingly, we find K to be nearly constant over the depth of the column, but $\tan\delta$ shows some variation (Supplementary Fig. 2b,c). Thus, the the rise of the σ_{rr} with z will be rapid, but not precisely exponential, and it will depend on the dimensions of the Couette cell; this is indeed reflected in the results of refs 11,12, where the fitted values of K and δ are found to vary with the fill height H. Nevertheless, the assumption of constant $\tan\delta$ is a rough first approximation that gives the correct qualitative variation of σ_{rr} with z.

Supplementary References

[1] Savage, S. B. & Hutter, K. The motion of a finite mass of granular material down a rough incline. *J. Fluid Mech.* **199**, 177–215 (1989).

[2] GDR MiDi. On dense granular flows. *Eur. Phys. J. E* **14** (2004).

[3] Lu, K., Brodsky, E. E. & Kavehpour, H. P. Shear-weakening of the transitional regime for granular flow. *J. Fluid Mech.* **587**, 347–372 (2007).

[4] Walker, D. M. An approximate theory for pressures and arching in hoppers. *Chem. Engng Sci.* **21**, 975–997 (1966).

[5] Cowin, S. C. The theory of static loads in bins. *Trans. ASME E: J. Appl. Mech.* **44**, 409–412 (1977).

[6] Ovarlez, G., Kolb, E. & Clément, E. Rheology of a confined granular material. *Phys. Rev. E* **64**, 060302 (2001).

[7] Nedderman, R. M. *Statics and Kinematics of Granular Materials* (Cambridge University Press, Cambridge, 1992).

[8] Sundaram, V. & Cowin, S. C. A reassessment of static bin pressure experiments. *Powder Technol.* **22**, 23–32 (1979).
